# Electrolytic lesions of the bilateral ventrolateral orbital cortex not only directly reduce depression-like behavior but also decreased desperate behavior induced by chronic unpredicted mild stress in rats

**DOI:** 10.1186/s12868-021-00677-6

**Published:** 2021-11-24

**Authors:** Zheng Chu, Wei Han, Peng Liu, Fei Liu, Gang Lei, Lisha Deng, Liu Yang, Yonghui Dang

**Affiliations:** 1 College of Medicine and Forensics, Xi’an Jiaotong University Health Science Center, 76 West Yanta Road, Xi’an, Shaanxi 710061 People’s Republic of China; 2 Department of Forensic Medicine, Xuzhou Medical University, Xuzhou, Jiangsu People’s Republic of China; 3 Department of Pharmacology and Toxicology, Institute of Basic Medicine Science, Xi’an Medical University, Xi’an, Shaanxi People’s Republic of China; 4 Clinical Research Center of Shaanxi Province for Dental and Maxillofacial Diseases, College of Stomatology, Xi’an Jiaotong University, Xi’an, Shaanxi People’s Republic of China; 5 Key Laboratory of the Health Ministry for Forensic Medicine, Xi’an Jiaotong University Health Science Center, Xi’an, Shaanxi People’s Republic of China; 6 Key Laboratory of Shaanxi Province for Forensic Medicine, Xi’an Jiaotong University Health Science Center, Xi’an, Shaanxi People’s Republic of China; 7 State Key Laboratory for Manufacturing Systems Engineering, Xi’an, Shaanxi People’s Republic of China

**Keywords:** Ventrolateral orbital cortex, Depression, Anxiety, Aggression, Chronic unpredicted mild stress

## Abstract

**Background:**

Previous studies have revealed that ventrolateral orbital cortex (VLO) may play an important role in the regulation of emotional behavior. However, it is not known what effect VLO damage will have on emotion regulation.

**Results:**

Data showed that damage of VLO increased the anxiety-like behavior in open field test and elevated plus maze, and decreased the depressive behavior in forced swimming test and learned helplessness test. Besides, the impulsive aggressive behaviors were also increased while the attack latency decreased after VLO lesion. What’s more, damage of VLO decreased depressive behaviors induced by chronic unpredicted mild stress in rats.

**Conclusions:**

These results suggest that the integrity of VLO plays an important role in emotional regulation, and the damage of VLO may inhibit the development of depression-like behavior.

## Background

The prefrontal cortex (PFC) plays a critical role in the generation and regulation of cognition and emotion [1, 2]. The abnormality of the PFC has been reported in various psychiatric disorders, such as depression, anxiety, schizophrenia and personality disorder [3–6]. The orbitofrontal cortex (OFC), as a key part of the PFC (Brodmann area 10, 11, 12, 47), is a subdivision of the frontal lobe and related to many neuroanatomical structures that are directly involved in emotions and executive processes, such as the hippocampus, amygdala, ventral striatum, anterior cingulate gyrus, hypothalamus and medial temporal region [7–10]. So far, most researches on OFC about its role in mental disorders regard it as a entirety. Human image studies have indicated that orbitofrontal abnormalities engage in the pathological process of many psychiatric diseases, such as depression, bipolar disorder and impulsive aggression [11–14]. Besides, human and primate researches prompt that OFC lesion results in defects in emotional and cognitive processing [14–17]. More studies on rodents confirm the important role of OFC in the generation and development of cognition and emotion [18, 19].

The ventrolateral orbital cortex (VLO) is one of the major divisions of the OFC. Our previous studies indicated that VLO may play an important role in the modulation of depressive disorder: microinjection of valproic acid into the VLO exerts an antidepressant-like effect [20]. However, the function of VLO as a potential emotion-regulating nucleus has not been evaluated. Therefore, in the present study, we will detailedly evaluate the neurobehavioral changes when the VLO is damaged by electric current and hope to advance the understanding of the potential role of VLO in emotional modulation.

## Methods

### Animals and experimental scheme

Male Sprague–Dawley rats (200–300 g) were provided by the Experimental Animal Center of Xi’an Jiaotong University. The rats were housed four per cage with a 12-h light/dark cycle (lights on at 7:00 a.m.), free to food and water. The study was approved by the Institutional Animal Care Committee of Xi’an Jiaotong University and all methods were performed in compliance with the relevant guidelines and regulations. All efforts were made to minimize the amount of animal use and the pain they suffered. All animals were acclimated to the environment and handled for 1 week before the start of the experiment.

**Fig. 1:**
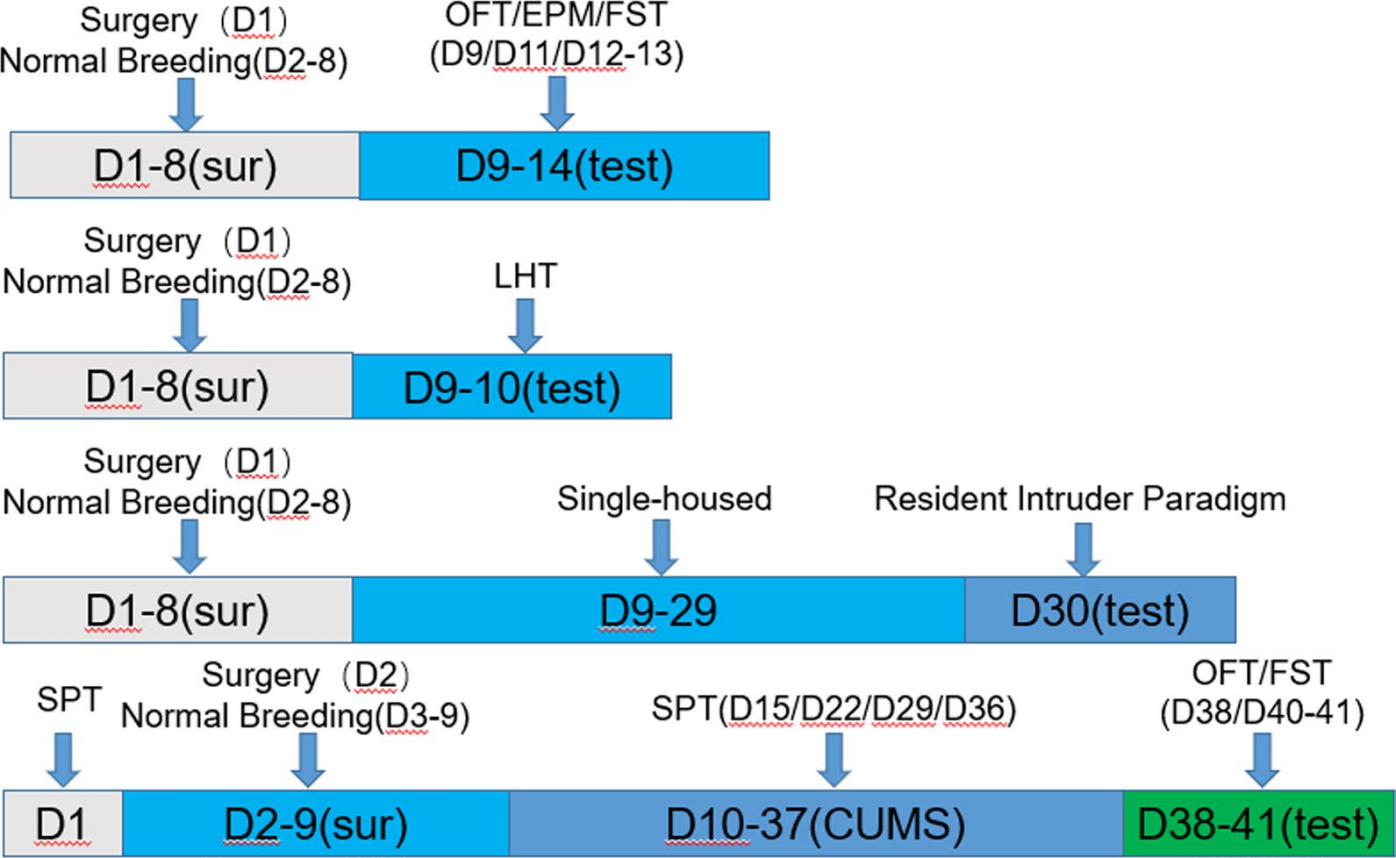
Experimental design

In this study, four cohorts of rats were used. Each cohort included 10 rats. A cohort of rats were used to do open filed test (OFT), elevated plus maze (EPM) and forced swimming test (FST). Then, another cohort of rats were used to perform learned helplessness test (LHT). Besides, a cohort of rats were used to carry out resident intruder paradigm-induced aggression test. Furthermore, a cohort of rats were used for chronic unpredictable mild stress (CUMS) procedure and subsequent behavioral experiments, such as OFT, FST and sucrose preference test (SPT). Except that the resident intruder paradigm-induced aggression test was performed from 19:00 to 23:00, other tests were completed from 9:00 to 12:00. The detailed experimental design is described in Fig. 1.

### Electrical lesion surgery

The operations were performed on a small-animal brain stereotactic instrument (RWD, Shenzhen, China) under anesthetized with intraperitoneal (i.p.) injection of 50 mg/kg pentobarbital sodium (SCRC, Shanghai, China). For bilateral VLO injury, a concentric stainless-steel microelectrode (diameter 0.2 mm, Stoelting, USA) was inserted into the VLO (3.2 mm posterior to bregma, ± 2.0 mm lateral to bregma, 4.6 mm from the cortical surface), and then a current of 400 μA was given for 30 s. After rats woke up from anesthesia, they received sodium penicillin (0.2 million units/day, i.p.) for 5 consecutive days to prevent wound and intracranial infection. Compared to electrical lesion (EL) group, the sham group rats received the same surgical treatment except for the electric current. All rats were allowed to recover for a week.

### Behavioral experiment

#### Open field test

To assess the locomotor activity ability and anxiety-like behavior of rats, an instrument consisted of a square plexiglass box of 100 cm × 100 cm and 40 cm high was used to measure movement distance and activity track of rats. The OFT protocol was carried out as previously described [21]. Animals were initially placed in the center of the box and allowed to explore freely for 60 min. A video-computerized tracking system (SMART, Panlab SL, Barcelona, Spain) was used to analyze the trajectory of each rat. The OFT was conducted under dim lighting conditions and boxes were cleaned with 75% alcohol to erase the smell between different animals.

#### Elevated plus maze

The EPM is an experiment aimed to investigate the anxiety state of animals by the conflict between animals' desire to explore new environments and their fear of heights and open arms. The EPM consisted of two open arms (50 cm × 10 cm) and two closed arms (50 cm × 10 cm × 20 cm) with an open roof, and the arms intersected at the central square (10 cm × 10 cm). There is a shelf under arms to raise the maze by 50 cm. The rats were placed in the central square with their heads facing open arms at the beginning of the test, and their performances were recorded for 6 min with a video system for later analysis by experimenters who were blind to the treatment of rats. The number of entries to the open/closed arms and the time spent in open/closed arms was counted. An entry was defined as four paws of the rat were placed in the arm [22].

#### Forced swimming test

The FST was performed as described previously [20] which consisted of two phases: pre-test (day 1) and test (day 2). On pre-test day, rats were placed into a plexiglass cylinder (25 cm diameter, 45 cm height) filled with 22–23 °C water, 25 cm deep for 10 min and then immediately removed from the barrel, dried and returned to their cages. 24 h later, the rats were placed in the cylinder again for 6 min, and their performances were recorded with a video system for later analysis by experimenters who were blind to the treatment of rats. These videos were used to calculate the immobility time rats spent in the water. Immobility was defined as only the action necessary to keep afloat [23].

#### Learned helplessness test

The LHT is an experiment aimed to observe the “give up” depression-like behavior when animals are exposed to incontrollable trauma. In this test, two commercial shuttle boxes (Med Associates, St. Albans, Vermont, USA) equipped with automatic door to separate two compartments of equal size were used [24]. The front, back, and ceiling of the shuttle box are transparent plexiglass, and the sides are aluminum. Underneath are stainless-steel bars with 5 mm in diameter and spaced 1.1 cm apart. Both boxes were housed in sound-attenuated chambers. Briefly, the LH procedure contained two phases: training phase (day 1) and active avoidance test phase (day 2). During training phase, rats were placed in the illuminated box and received inescapable footshocks (IES) in the shuttle box with the door opening (60 trials, 0.8 mA intensity, 15 s duration, average 15 s intertrial interval). The IES was accompanied by a 60 Hz noise which was considered as the conditioned stimulus (CS) while the inescapable footshocks was defined as unconditioned stimulus (US). Twenty-four hours after training, rats were placed into the box again, and the CS was presented for 5 s followed by presentation of CS and US for 25 s at the same time. At the end of the trial, the CS and the US were automatically turned off. When the animal moves into another compartment within 5 s and avoided the US, it is recorded as a “avoidance”. However, if the animal failed to avoid the US, it can also escape it within 25 s which is recorded as an “escape”. Between each trial, there was an interval of mean 30 s. Each animal received 30 trials.

#### Resident intruder paradigm-induced aggression test

The resident intruder test was performed in the early hours of the dark phase (from 19:00 to 23:00) when rats express more active. The encounter was conducted with no light. After surgery, resident rats were single-housed for 3 weeks in a big cage (90 cm × 60 cm × 25 cm). Because the territory consciousness is strongly based on olfactory cues, the bedding was not replaced in the last week. When the tests began, a smaller male rat was introduced into the resident cage, and their behaviors were recorded for 30 min by an infrared camera. The number of attacks and the time that the resident rat spent to perform its first attack were scored later by an experimenter blind to surgery conditions. The following behaviors were defined as attacks: bites, clinch, chase, wrestling, upright posture by the resident and keep down.

### Sucrose preference test

The SPT is used to test for anhedonia which is one of the core symptoms of depression and a sign of the success of the CUMS model [25]. This experiment was divided into training and testing stages. For training period, the rats were given two bottles for 48 h, one of which contained 1% (w/v) sucrose solution and the other holding tap water.

During testing period, rats were banned from water for 16 h and then allowed to drink two bottles for 1 h. During the CUMS procedure, SPT only was performed on every Saturday. The sucrose preference index was calculated using the following formula: sucrose preference = sucrose intake (g)/(sucrose intake (g) + water intake (g).

### Chronic unpredictable mild stress model

The CUMS procedure was performed as described with slightly modification [26]. In brief, it was a random 4-week plan with various stressors: 45° tilting cage; continuous illumination; water deprivation; empty bottle; group-housed; reversal illumination; stroboscopic lighting; tail clamp; wet cage; packing deprivation; restraint; food deprivation; dark deprivation; wet cage; packing deprivation. The duration of various stressors, and even of the same stressor was different. Besides, the order in which various stresses were administered was pseudo-random (Table 1). During the CUMS procedure, animals were divided into four groups: sham/group-housed(GH) group, sham/CUMS group, EL/group-housed group and EL/CUMS group. And group-housed animals were placed in a separate room with normal breeding.

**Table 1:** Chronic unpredictable mild stress procedure

	Monday	Tuesday	Wednesday	Thursday	Friday	Saturday	Sunday
First week	08:00 tilted cage	07:00 illumination	10:00–11:00 empty bottle	07:00 reversal illumination	10:00 tail clamp	10:00–11:00 SPT	10:00 flat cage
	21:00 flat cage	15:00 deprive water	11:00 full bottle	13:00 monoculture	14:00 deprive water	11:00 provide water	wet cage
	illumination		18:00 two in a cage	13:00–19:00 stroboscopic lighting		15:00 tilted cage	
Second week	08:00 empty cage	08:00 restraint (2 h)	07:00	13:00 monoculture	14:00 deprive water	10:00–11:00 SPT	12:00 empty cage
			Reversal illumination			11:00 provide water	
	18:00 clear bedding	17:00 deprive food	13:00–19:00 stroboscopic lighting	13:00 monoculture		17:00 wet cage	
			17:00 provide food				
Third week	08:00 clear bedding	10:00–19:00 two in a cage	19:00 provide water	08:00 flat cage	08:00 restraint (2 h)	10:00–11:00 SPT	08:00 replace bedding
	10:00 tail clamp	19:00 deprive water	19:00 tilted cage	reversal illumination	14:00 deprive water	11:00 provide water	17:00 deprive food
	18:00 illumination			13:00–19:00 stroboscopic lighting		15:00 wet cage	
Fourth week	10:00 provide food	10:00–11:00 empty bottle	08:00 restraint (2 h)	07:00 reversal illumination	13:00 flat cage	10:00–11:00 SPT	
		11:00 provide water		11:00 replace bedding			
	10:00 provide food	19:00 illumination	08:00 restraint (2 h)	11:00–19:00 stroboscopic lighting	13:00 flat cage	10:00–11:00 SPT	

### Histology

**Fig. 2:**
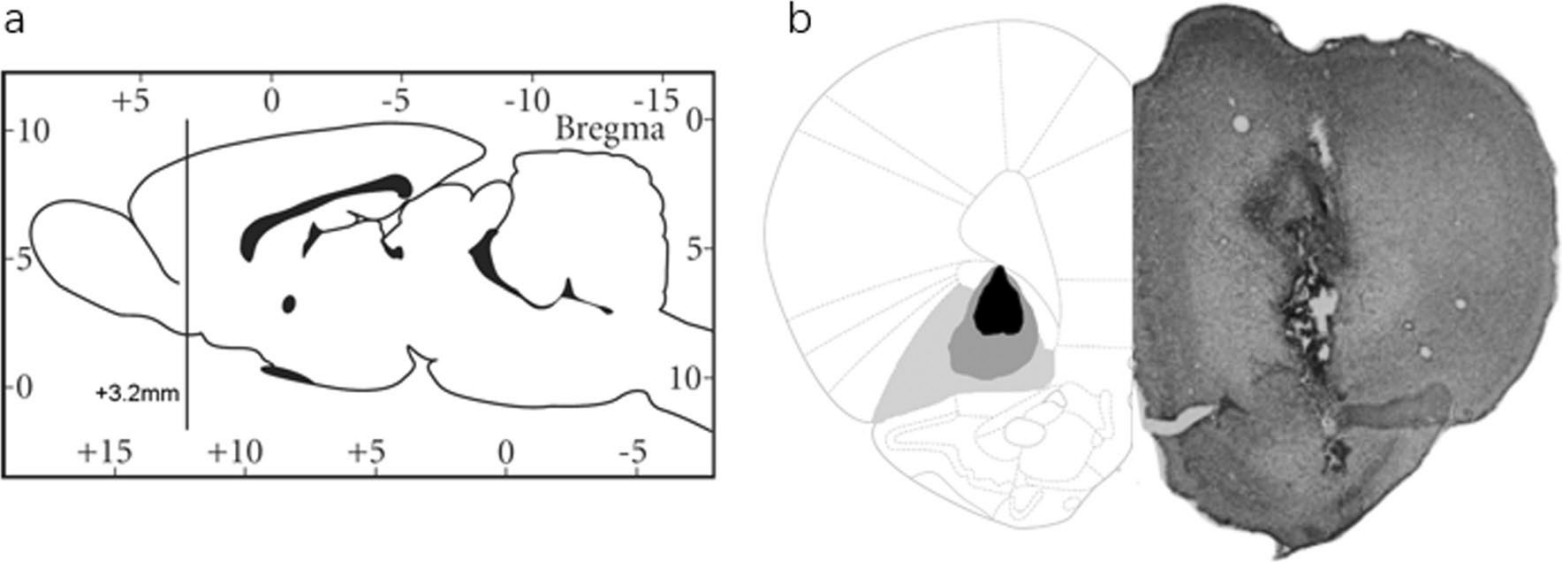
Histological verification of electrolytic lesions of the bilateral VLO of rat, using coordinates on the sagittal and coronal planes in millimeters (from Bregma) according to George Paxinos and Charles Watson (2006). **A** Diagram of rat brain sagittal section showing the position of VLO. **B** Left: Diagram of rat brain sagittal sections showing the extent of the bilateral electrolytic lesions in the VLO. The VLO is highlighted in light gray. The minimum lesion extent is shown in black, and the maximum extent is shown in dark gray. Right: Photomicrographs of a coronal section from a representative rat

One day later after the behavioral experiments were performed, the locations of electric lesion were conformed. Under deep anesthesia with 100 mg/kg sodium pentobarbital, i.p., rats were firstly transcardially perfused with 150 ml of 0.01 M phosphate-buffered saline (PBS, pH 7.4) followed by 400 ml of 4% paraformaldehyde. The brains were then separated and fixed in 4% paraformaldehyde solution at 4 °C. Five days later, the brain were immersed in 30% (w/v) 0.1 M PB (pH 7.4) sucrose solution for 3 days at 4 °C, and the solution was changed once in the middle. Then, the brains were cut into 30 μm slices on a cryostat microtome. The slices were stained with cresyl viole and the lesion locations were verified through visual inspection. Only data from rats with the correct injection location were included in the data analysis (Fig. 2).

### Statistical analysis

Statistical analyses were performed using the commercially available software GraphPad Prism 6.0. All data were expressed as the mean ± SEM. The normality of distribution was evaluated using the D’Agostino & Pearson test (p > 0.05). Data were analyzed using unpaired Student’s *t* test for parametric test and Mann–Whitney test for nonparametric test between two groups and two-way analysis of variance (ANOVA) with rearing condition and operative methods as between-subject factors. Student’s t test was used to analyze difference when a significant interaction didn’t exist. p < 0.05 were considered to be statistically significant.

## Results

### Effect of electrolytic lesions of the bilateral VLO on OFT and EPM

**Fig. 3:**
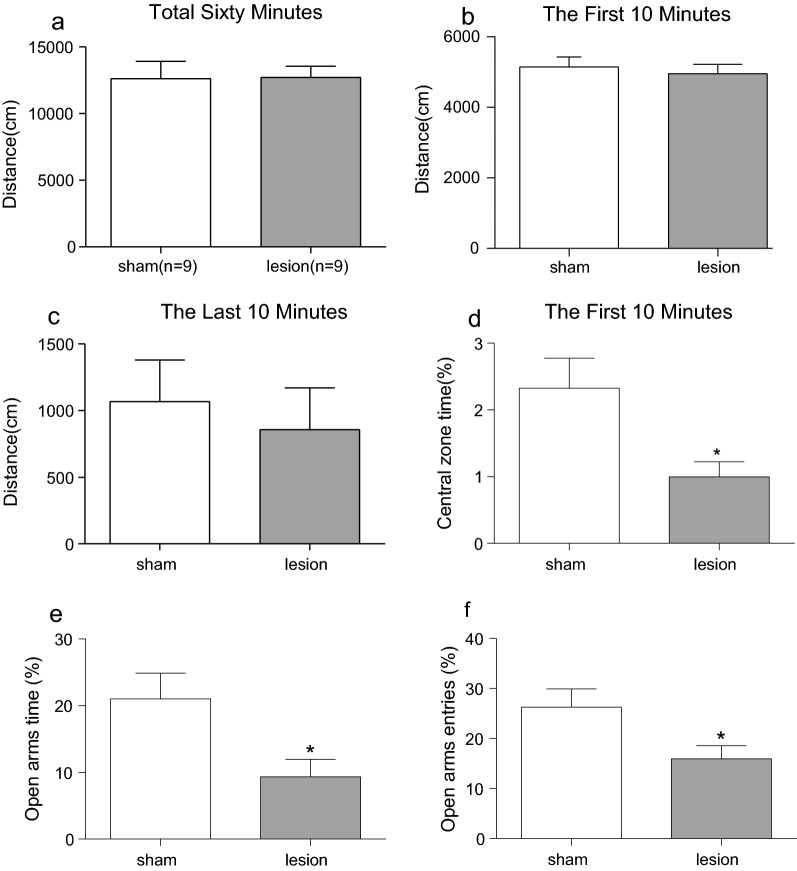
Effect of electrolytic lesions of the bilateral VLO on OFT and EPM. **A** The total distance rats spent in 60 min; **B** The distance rats spent in first 10 min; **C** The distance rats spent in last 10 min; **D** The percentage of time spent in the central zone area during first 10 min. **E** The percentage of time spent in the open arms; **F** The percentage of numbers of entries to the open arms; Unpaired Student’s *t*-test analysis was used, compared between lesion and sham groups, *p < 0.05

As shown in Fig. 3A, in the OFT, there was no significant differences between the two groups in the total distance (p > 0.05). Besides, no significant difference was observed between the EL and sham rats in the first and last 10 min (p > 0.05, Fig. 3B; p = 0.639, Fig. 3C). What’s more, compared with sham group, the time spent in central zone significantly decreased in VLO-lesion group during the first 10 min (p < 0.05, Fig. 3D).

In the EPM, lesion of bilateral VLO significantly decreased the time spent in open arms compared to the sham group (p < 0.05, Fig. 3E). The numbers of entries to the open arms were also significantly declined (p < 0.05, Fig. 3F).

### Effect of electrolytic lesions of the bilateral VLO on FST and LHT

**Fig. 4:**
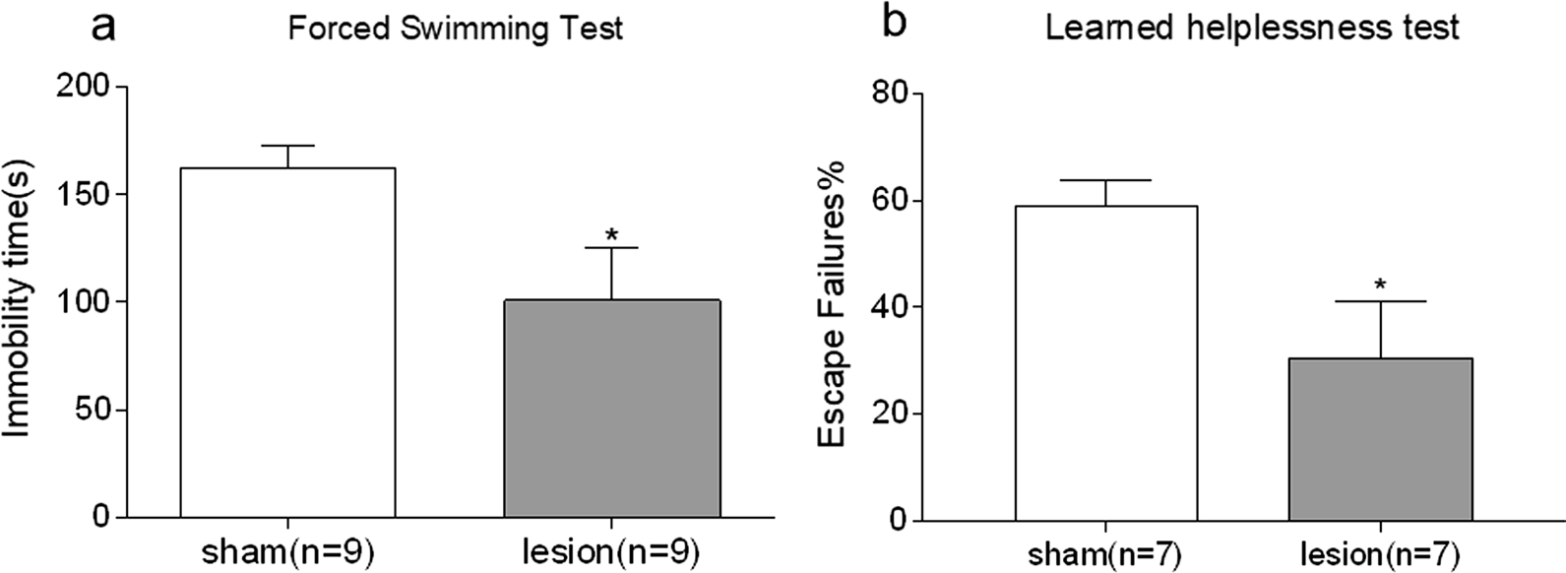
Effect of electrolytic lesions of the bilateral VLO on FST and LHT. **A** The immobility time spent in FST during 6 min; **B** The percentage of numbers of escape failure; Unpaired Student’s t-test analysis was used, compared between lesion and sham groups, *p < 0.05

As shown in Fig. 4A, electrolytic lesion of bilateral VLO significantly decreased the immobility time during total 6 min (p < 0.01). This anti-depressive behavior found in FST was reproduced in LHT. The percentage of escape failures was found to be significantly reduced with rats in EL group (p < 0.05, Fig. 4B).

### Effect of electrolytic lesions of the bilateral VLO on resident intruder paradigm-induced aggression

**Fig. 5:**
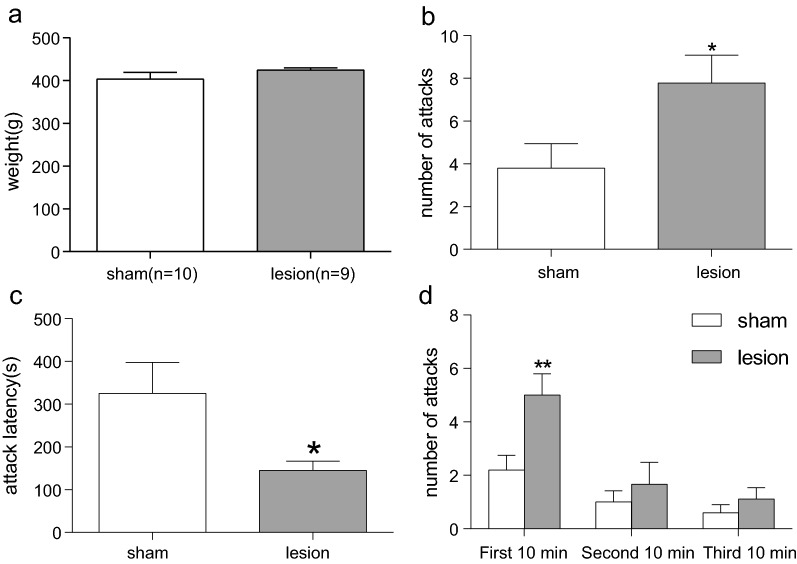
Effect of electrolytic lesions of the bilateral VLO on resident intruder paradigm-induced aggression. **A** The body weight; **B** The number of attack; **C** The attack latency; **D** The number of attacks every 10 min. Unpaired Student’s *t*-test analysis was used, compared between lesion and sham groups, *p < 0.05, **p < 0.01

Firstly, as shown in Fig. 5A, there was no significant difference between two groups about their weight (403.9 ± 15.33, 424.9 ± 5.05, p > 0.05). The damage of VLO significantly increased the number of attacks (p < 0.05, Fig. 5B) during total 30 min and decreased the attack latency (p < 0.05, Fig. 5C). Then, the numbers of attacks during every 10 min were analyzed. As seen in Fig. 5D, the damage of VLO significantly increased the number of attacks during first 10 min (p < 0.01), while there was no significantly difference between the two groups during the second (p > 0.05) and third 10 min(p > 0.05).

### Effect of electrolytic lesions of the bilateral VLO on OFT in CUMS model

**Fig. 6:**
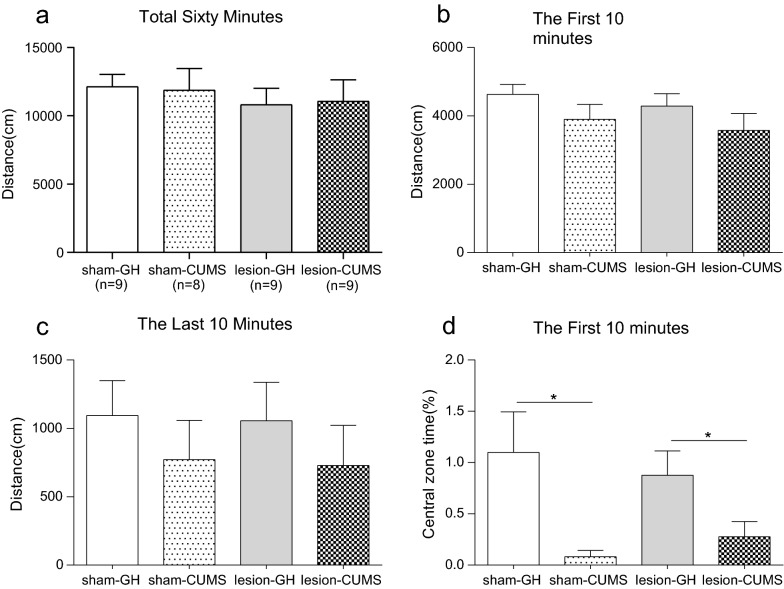
Effect of electrolytic lesions of the bilateral VLO on OFT in CUMS model. **A** The total distance rats spent in 60 min; **B** The distance rats spent in first 10 min; **C** The distance rats spent in last 10 min; **D** The percentage of time spent in the central zone area during first 10 min. Two-way ANOVA and unpaired Student’s *t*-test analysis was used, *p < 0.05

As shown in Fig. 6A, in the OFT, both rearing condition (F = 0.001, p > 0.05) and operative method (F = 0.654, p > 0.05) had no statistical difference on the immobility time. The same results were found in the first and last 10 min (Fig. 6B and C). However, when analyzing the central zone time in first 10 min, rearing conditions had significant effect on the central zone time (F = 9.763, p < 0.01) while operative method shown no significant effect (F = 0.000, p > 0.05), as shown in Fig. 6D. For sham groups, the central zone time of GH rats was significantly higher than CUMS rats (p < 0.05). For lesion groups, the central zone time of GH rats was also significantly higher than CUMS rats (p < 0.05).

### Effect of electrolytic lesions of the bilateral VLO on SPT and FST in CUMS model

**Fig. 7:**
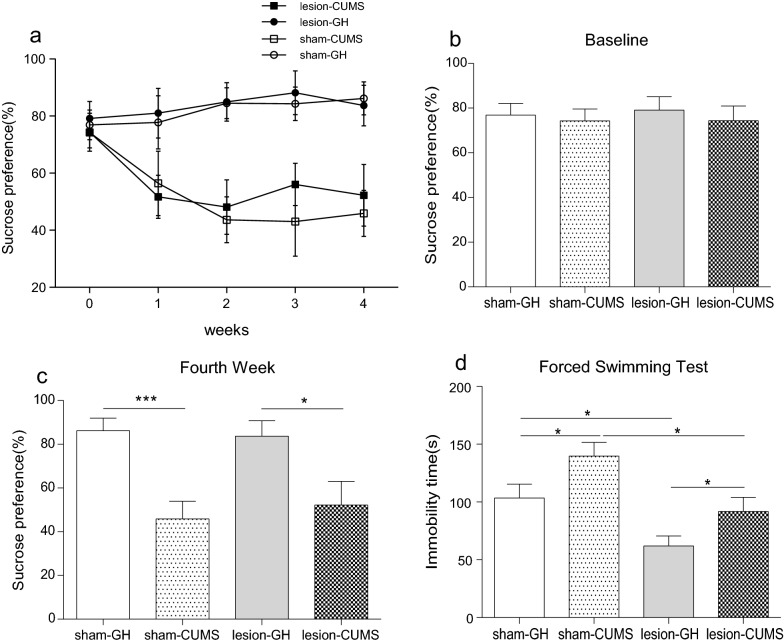
Effect of electrolytic lesions of the bilateral VLO on SPT and FST in CUMS model. **A** The total four weeks; **B** Baseline of SPT; **C** The fourth week of SPT; **D** The immobility time in FST. Two-way ANOVA and unpaired Student’s *t*-test analysis was used, *p < 0.05, ***p < 0.001

In SPT, the relative sucrose preference at baseline shown no significant differences among four groups before CUMS started (Fig. 7B). After CUMS protocol and surgery operation, rearing condition started to have effects on the relative sucrose preference, while no significant effect was found in operative method. In the fourth week, a significant decrease of relative sucrose preference was found in CUMS rats compared with GH rats, indicating that the core symptom of depression—anhedonia was successfully derived (Fig. 7C). But there was no difference in sucrose preference between different surgical methods (Fig. 7C).

In FST, both rearing condition (F = 8.458, p < 0.01) and operative method (F = 15.39, p < 0.001) had statistical differences on the immobility time. But no significant differences of interaction effect were found (F = 0.081, p > 0.05). Consistent with the above result, VLO-lesion significantly decreased the immobility time compared between GH groups (Fig. 7D, p < 0.05).In lesion groups, CUMS increased the immobility time compared with GH rats, indicating that the anti-depressive effect of VLO-lesion could not preventing the development of depression-like behavior after four-weeks CUMS protocol (p < 0.05). However, in CUMS groups, VLO-lesion indeed decreased the immobility time, suggesting that the damage of VLO reduced depressive behaviors induced by CUMS procedure (p < 0.05), while CUMS could induce depressive behavior in sham groups (p < 0.05).

## Discussion

Results from the present study indicated that electrolytic lesions of the bilateral VLO increased the anxiety-like behavior in OFT and EPM but significantly decreased the depressive behavior in FST and LHT. Besides, VLO-lesion increased the impulsive aggressive behavior in resident intruder paradigm-induced aggression test. However, damage of the bilateral VLO ameliorated depressive behaviors induced by CUMS in rats.

The OFC is considered as a key node in the emotional circuit of the brain. About its role in regulation of emotion, there are two main theories. One is that OFC manages emotion and enhances the behavioral flexibility through inhibitory control [27, 28]. The other contributes its role to the value of assessment based on the basis of current motivational states [29, 30]. The OFC is a heterogeneous cortical region composed of several regions that can be differentiated by their cellular structure, neurochemical characteristics, and their internal and external connections with other brain regions [31–33]. Our previous suggested that both histone methylation and DNA methylation in VLO were involved in regulation of depressive behavior [20, 34]. So we designed these experiment to evaluate the effect of VLO damage in the modulation of emotion.

### Electrolytic lesions of the bilateral ventrolateral orbital cortex increase anxiety-like behavior and impulsive aggression, but decrease depression-like behavior in rats

In this study, we initially demonstrated that electrolytic lesions of the bilateral VLO didn’t affect the athletic ability of rats which provided the basis for other behavioral tests. Meanwhile, the decreased time spent in central area indicated the electrolytic lesions of the bilateral VLO played an anxiety-inducing role. This effect was confirmed by the EPM in which VLO-lesion rats spent less time in the open arms. All these results suggested that the VLO may play an anti-anxiety role. In a human image study, gray matter volume of the OFC was associated with increased optimism, which in turn was associated with reduced anxiety [19], on the contrary, the reduced volume of the OFC was associated with anxiety in patients with panic disorder [35]. On the other hand, anxiety elicited the disruption of the OFC by reducing the firing rat of spontaneously active neuronal subpopulations [36]. Combined with the results of our experiment, we guessed that VLO may play an anxiety-inducing role by integrating external information input and internal value assessment.

Our result of the FST in which VLO-lesion rats performed less immobility time in water indicated that damage of the VLO may lead to antidepressant-like behavior. This result was confirmed by the response observed in LHT in which VLO-lesion group shown a lower number of “escape failures”. Damage to the VLO, whether in water that cannot escape or in a shuttle box that cannot avoid the shock, caused the rats to give up earlier. In a prospective cohort study included 254 Vietnam War veterans, damage of the OFC may lead to a reduction in psychiatric symptoms, possibly due to the interruption of normal emotional responses [37]. Some studies prompted that patients with depression have a lower volume in OFC [38, 39]. However, Opel and Redlich found that patients with major depressive disorder (MDD) had significantly increased activation in the medial OFC when they appeared to negative stimuli [40]. In contrary, damage in this area is associated with a reduction in depressive symptoms in patients with MDD [41]. The seemingly contradictory conclusions from different studies may be related to the fact that subregions of the OFC are not distinguished. From our test results, the integrity of VLO is necessary for the formation of depression-like behavior.

Then, considering the close relationship between aggression, depression and anxiety, we performed a resident intruder paradigm-induced aggression test. Results demonstrated that after electrolytic lesions of VLO, rats expressed higher numbers of attacks and lower attack latency. In daily life, emotional stress usually manifests as depression, anxiety and anger which tends to evolve into verbal or behavioral aggression. The thalamic nucleus submedius (Sm)-VLO-periaqueductal gray (PAG) is a pain modulation pathway studied in our laboratory which forms a loop consisting of spinal cord/trigeminal nerve-Sm-VLO-PAG- spinal cord/trigeminal nerve in the central nervous system [42]. PAG plays an important role in regulating various biological functions (such as stereotypic defense and triggering of reproductive behavior, pain, anxiety, urinary, cardiovascular and respiratory activities) [43, 44]. c-Fos researches shown that PAG was activated during the attack and participated in the attack [45–47]. However, the degree of PAG activation was inhibited in the highly aggressive species compared to the less aggressive species [48–50]. The drug or electrical activation of VLO inhibited tail flick reflex and jaw opening reflex in a dose or intensity dependent manner, and these effects could be eliminated by damaging PAG or injecting GABA into PAG [51–53]. In this study, we suspect that one of the mechanisms by which VLO participates in the regulation of aggressive behavior may be the downward regulation of PAG.

What’s more, patients with neurodegenerative diseases that affect OFC usually show a syndrome of emotional retardation [54]. In contrast, patients with neurodegeneration (such as Parkinson's disease and Huntington's disease) who initially spare OFC often exhibit symptoms of depression, irritability, and anxiety, indicating that both normal emotional experience and excessive emotional pathological experience (including depression, anxiety and aggression) need a fully functional OFC [55, 56]. Our results shown that as a key part of the OFC, the integrity of VLO is also important for the normal emotional experience.

### Electrolytic lesions of the bilateral ventrolateral orbital cortex reduced depressive behaviors induced by chronic unpredicted mild stress in rats

Above experiments revealed that the damage of VLO in adulthood could immediately lead to the change of emotional behavior, but the effect of subsequent stress events on emotional behavior has not been known. So, we designed an experiment to investigate the subsequent influence on the rat CUMS model of VLO lesion. Firstly, the present results showed that the destruction of VLO didn’t increase the anxiety-like behavior of rats induced by CUMS procedure (sham-CUMS vs lesion-CUMS). Secondly, the present results showed that VLO damage decreased the degree of depressive behavior (sham-CUMS vs lesion-CUMS) without preventing its formation (lesion-GH vs lesion-CUMS). These phenomena suggested that the damage of VLO may play an antidepressant role, but it cannot completely prevent the formation of depression-like behavior. After long-term stress events, the rats still had depression-like behavior, but the degree of depression was reduced compared with the rats without damage of VLO.

In this part, long-term emotional behavioral changes after VLO damage were more considered to be related to the deficit of reward evaluation and update. The function of accumulated reward storage and renewal based on positive and negative events has been suggested to be the responsibility of the OFC [57]. The delay or inability of behavior change after encountering adverse stress with orbitofrontal damage might be the result of the inability of correctly evaluating reinforcement factors. Here, we speculated that VLO also played an important role in the process of reward assessment, and the inability to evaluate and store adverse stress cues after VLO damage reduced the extent of behavior changes caused by specific reinforcement cues. Specific to present study, our data indicated that VLO played an important role in the assessment of chronic unpredictable mild stresses and its incomplete structure and function would lead to resistance to depression.

There are also some limitations should be noticed in our experiment. Firstly, time spent in center of the OFT was reduced by the lesion in the first cohort but the result was not reproduced in the CUMS experiment. We think it maybe that some regions of brain compensate for the modulation of anxiety behavior after VLO damage. It is also possible that CUMS procedure has a deep impact on anxiety-like behavior, so the anxiety inducing effect of VLO damage is insignificant compared with CUMS treatment. Besides, anxiety and depression are usually comorbid. But our results shown that electrolytic lesions of the bilateral VLO increased the anxiety-like behavior but significantly decreased the depressive behavior. This phenomenon is very interesting and worthy of further study. Lastly, electrical injury is a precise method but it can destroy nerve fibers passing through.

## Conclusion

In conclusion, the present study suggests that VLO plays an important role in the regulation of emotion. While the electrolytic lesions of VLO increased the anxiety-like and aggressive behavior, the depressive behaviors were inhibited both in FST and LHT. Besides, VLO damage had no effect on anxiety-like behavior induced by CUMS in rats, but **decreased** the degree of depressive behavior without preventing its formation. The integrity of VLO is necessary for normal affective experience, and further studies need to explore the mechanism how VLO regulates emotions.

## Data Availability

The datasets used and/or analyzed during the current study are available from the corresponding authors on reasonable request.

## Abbreviations

CUMS: Chronic unpredictable mild stress
EPM: Elevated plus maze
FST: Forced swimming test
i.p.: Intraperitoneally
LHT: Learned helplessness test
MDD: Major depressive disorder
OFC: Orbitofrontal cortex
OFT: Open filed test
PAG: Periaqueductal gray
PFC: Prefrontal cortex
Sm: Thalamic nucleus submedius
SPT: Sucrose preference test
VLO: Ventrolateral orbital cortex

## References

[1] Dixon ML, Thiruchselvam R, Todd R, Christoff K (2017). Emotion and the prefrontal cortex: an integrative review. Psychol Bull.

[2] Nyberg L (2018). Cognitive control in the prefrontal cortex: a central or distributed executive?. Scand J Psychol.

[3] Levkovitz Y, Harel EV, Roth Y, Braw Y, Most D, Katz LN, Sheer A, Gersner R, Zangen A (2009). Deep transcranial magnetic stimulation over the prefrontal cortex: evaluation of antidepressant and cognitive effects in depressive patients. Brain Stimul.

[4] d’Arbeloff TC, Kim MJ, Knodt AR, Radtke SR, Brigidi BD, Hariri AR (2018). Microstructural integrity of a pathway connecting the prefrontal cortex and amygdala moderates the association between cognitive reappraisal and negative emotions. Emotion.

[5] Orlov ND, Tracy DK, Joyce D, Patel S, Rodzinka-Pasko J, Dolan H, Hodsoll J, Collier T, Rothwell J, Shergill SS (2017). Stimulating cognition in schizophrenia: a controlled pilot study of the effects of prefrontal transcranial direct current stimulation upon memory and learning. Brain Stimul.

[6] McClure MM, Barch DM, Romero MJ, Minzenberg MJ, Triebwasser J, Harvey PD, Siever LJ (2007). The effects of guanfacine on context processing abnormalities in schizotypal personality disorder. Biol Psychiatry.

[7] Rolls ET (1996). The orbitofrontal cortex. Philos Trans R Soc Lond B Biol Sci.

[8] Berridge KC, Kringelbach ML (2008). Affective neuroscience of pleasure: reward in humans and animals. Psychopharmacology.

[9] Izquierdo A (2017). Functional heterogeneity within rat orbitofrontal cortex in reward learning and decision making. J Neurosci.

[10] Jackowski AP, Araujo Filho GM, Almeida AG, Araujo CM, Reis M, Nery F, Batista IR, Silva I, Lacerda AL (2012). The involvement of the orbitofrontal cortex in psychiatric disorders: an update of neuroimaging findings. Braz J Psychiatry.

[11] Spuhler K, Bartlett E, Ding J, DeLorenzo C, Parsey R, Huang C (2018). Diffusion entropy: a potential neuroimaging biomarker of bipolar disorder in the temporal pole. Synapse.

[12] Nam HY, Song SH, Kim SJ, Kwak IS, Kim IJ, Lee SB, Lee DW, Kim BS, Pak K, Kim YK (2011). Effect of dialysis on cerebral blood flow in depressive end-stage renal disease patients. Ann Nucl Med.

[13] McCabe C, Woffindale C, Harmer CJ, Cowen PJ (2012). Neural processing of reward and punishment in young people at increased familial risk of depression. Biol Psychiatry.

[14] Hermans EJ, Ramsey NF, van Honk J (2008). Exogenous testosterone enhances responsiveness to social threat in the neural circuitry of social aggression in humans. Biol Psychiatry.

[15] Maki-Marttunen V, Kuusinen V, Perakyla J, Ogawa KH, Brause M, Brander A, Hartikainen KM (2017). Greater attention to task-relevant threat due to orbitofrontal lesion. J Neurotrauma.

[16] Mansouri FA, Buckley MJ, Tanaka K (2014). The essential role of primate orbitofrontal cortex in conflict-induced executive control adjustment. J Neurosci.

[17] Kalin NH, Shelton SE, Davidson RJ (2007). Role of the primate orbitofrontal cortex in mediating anxious temperament. Biol Psychiatry.

[18] Orsini CA, Trotta RT, Bizon JL, Setlow B (2015). Dissociable roles for the basolateral amygdala and orbitofrontal cortex in decision-making under risk of punishment. J Neurosci.

[19] Rudebeck PH, Walton ME, Millette BH, Shirley E, Rushworth MF, Bannerman DM (2007). Distinct contributions of frontal areas to emotion and social behaviour in the rat. Eur J Neurosci.

[20] Xing B, Zhao Y, Zhang H, Dang Y, Chen T, Huang J, Luo Q (2011). Microinjection of valproic acid into the ventrolateral orbital cortex exerts an antidepressant-like effect in the rat forced swim test. Brain Res Bull.

[21] Zhao Y, Liu P, Chu Z, Liu F, Han W, Xun X, Dang YH (2015). Electrolytic lesions of the bilateral ventrolateral orbital cortex inhibit methamphetamine-associated contextual memory formation in rats. Brain Res.

[22] Zhao Y, Wang S, Chu Z, Dang Y, Zhu J, Su X (2017). MicroRNA-101 in the ventrolateral orbital cortex (VLO) modulates depressive-like behaviors in rats and targets dual-specificity phosphatase 1 (DUSP1). Brain Res.

[23] Zhao Y, Xing B, Dang Y-h, Qu C-l, Zhu F, Yan C-x (2013). Microinjection of valproic acid into the ventrolateral orbital cortex enhances stress-related memory formation. PLoS ONE.

[24] Hajszan T, Dow A, Warner-Schmidt JL, Szigeti-Buck K, Sallam NL, Parducz A, Leranth C, Duman RS (2009). Remodeling of hippocampal spine synapses in the rat learned helplessness model of depression. Biol Psychiatry.

[25] Liu F, Dong YY, Lei G, Zhou Y, Liu P, Dang YH (2021). HINT1 is involved in the chronic mild stress elicited oxidative stress and apoptosis through the PKC epsilon/ALDH-2/4HNE pathway in prefrontal cortex of rats. Front Behav Neurosci.

[26] Chen C, Dong Y, Liu F, Gao C, Ji C, Dang Y, Ma X, Liu Y (2020). A study of antidepressant effect and mechanism on intranasal delivery of BDNF-HA2TAT/AAV to rats with post-stroke depression. Neuropsychiatr Dis Treat.

[27] Roberts AC, Wallis JD (2000). Inhibitory control and affective processing in the prefrontal cortex: neuropsychological studies in the common marmoset. Cereb Cortex.

[28] Rudebeck PH, Saunders RC, Prescott AT, Chau LS, Murray EA (2013). Prefrontal mechanisms of behavioral flexibility, emotion regulation and value updating. Nat Neurosci.

[29] Howard JD, Kahnt T (2017). Identity-specific reward representations in orbitofrontal cortex are modulated by selective devaluation. J Neurosci.

[30] Kazama AM, Davis M, Bachevalier J (2014). Neonatal lesions of orbital frontal areas 11/13 in monkeys alter goal-directed behavior but spare fear conditioning and safety signal learning. Front Neurosci.

[31] Barbas H (2007). Flow of information for emotions through temporal and orbitofrontal pathways. J Anat.

[32] Price JL (2007). Definition of the orbital cortex in relation to specific connections with limbic and visceral structures and other cortical regions. Ann N Y Acad Sci.

[33] Bachevalier J, Machado CJ, Kazama A (2011). Behavioral outcomes of late-onset or early-onset orbital frontal cortex (areas 11/13) lesions in rhesus monkeys. Ann N Y Acad Sci.

[34] Xing B, Liu P, Xu W-j, Xu F-y, Dang Y-h (2014). Effect of microinjecting of 5-aza-2-deoxycytidine into ventrolateral orbital cortex on depressive-like behavior in rats. Neurosci Lett.

[35] Roppongi T, Nakamura M, Asami T, Hayano F, Otsuka T, Uehara K, Fujiwara A, Saeki T, Hayasaka S, Yoshida T (2010). Posterior orbitofrontal sulcogyral pattern associated with orbitofrontal cortex volume reduction and anxiety trait in panic disorder. Psychiatry Clin Neurosci.

[36] Park J, Wood J, Bondi C, Del Arco A, Moghaddam B (2016). Anxiety evokes hypofrontality and disrupts rule-relevant encoding by dorsomedial prefrontal cortex neurons. J Neurosci.

[37] Huey ED, Lee S, Lieberman JA, Devanand DP, Brickman AM, Raymont V, Krueger F, Grafman J (2016). Brain regions associated with internalizing and externalizing psychiatric symptoms in patients with penetrating traumatic brain injury. J Neuropsychiatry Clin Neurosci.

[38] Dusi N, Barlati S, Vita A, Brambilla P (2015). Brain structural effects of antidepressant treatment in major depression. Curr Neuropharmacol.

[39] Lai T, Payne ME, Byrum CE, Steffens DC, Krishnan KR (2000). Reduction of orbital frontal cortex volume in geriatric depression. Biol Psychiatry.

[40] Opel N, Redlich R, Grotegerd D, Dohm K, Zaremba D, Meinert S, Burger C, Plumpe L, Alferink J, Heindel W (2017). Prefrontal brain responsiveness to negative stimuli distinguishes familial risk for major depression from acute disorder. J Psychiatry Neurosci.

[41] Hamani C, Mayberg H, Stone S, Laxton A, Haber S, Lozano AM (2011). The subcallosal cingulate gyrus in the context of major depression. Biol Psychiatry.

[42] Tang JS, Qu CL, Huo FQ (2009). The thalamic nucleus submedius and ventrolateral orbital cortex are involved in nociceptive modulation: a novel pain modulation pathway. Prog Neurobiol.

[43] Rossi F, Maione S, Berrino L (1994). Periaqueductal gray area and cardiovascular function. Pharmacol Res.

[44] Zare A, Jahanshahi A, Rahnama'i MS, Schipper S, van Koeveringe GA (2019). The role of the periaqueductal gray matter in lower urinary tract function. Mol Neurobiol.

[45] Nelson RJ, Trainor BC (2007). Neural mechanisms of aggression. Nat Rev Neurosci.

[46] Lonstein JS, Stern JM (1997). Role of the midbrain periaqueductal gray in maternal nurturance and aggression: c-fos and electrolytic lesion studies in lactating rats. J Neurosci.

[47] Lonstein JS, Gammie SC (2002). Sensory, hormonal, and neural control of maternal aggression in laboratory rodents. Neurosci Biobehav Rev.

[48] Kollack-Walker S, Newman SW (1995). Mating and agonistic behavior produce different patterns of Fos immunolabeling in the male Syrian hamster brain. Neuroscience.

[49] Delville Y, De Vries GJ, Ferris CF (2000). Neural connections of the anterior hypothalamus and agonistic behavior in golden hamsters. Brain Behav Evol.

[50] Gammie SC, Nelson RJ (2001). cFOS and pCREB activation and maternal aggression in mice. Brain Res.

[51] Zhang S, Tang JS, Yuan B, Jia H (1997). Involvement of the frontal ventrolateral orbital cortex in descending inhibition of nociception mediated by the periaqueductal gray in rats. Neurosci Lett.

[52] Zhang YQ, Tang JS, Yuan B, Jia H (1997). Inhibitory effects of electrically evoked activation of ventrolateral orbital cortex on the tail-flick reflex are mediated by periaqueductal gray in rats. Pain.

[53] Zhang S, Tang JS, Yuan B, Jia H (1998). Inhibitory effects of electrical stimulation of ventrolateral orbital cortex on the rat jaw-opening reflex. Brain Res.

[54] Karageorgiou E, Miller BL (2014). Frontotemporal lobar degeneration: a clinical approach. Semin Neurol.

[55] Julien CL, Thompson JC, Wild S, Yardumian P, Snowden JS, Turner G, Craufurd D (2007). Psychiatric disorders in preclinical Huntington’s disease. J Neurol Neurosurg Psychiatry.

[56] Marsh L (2013). Depression and Parkinson’s disease: current knowledge. Curr Neurol Neurosci Rep.

[57] Fellows LK (2007). The role of orbitofrontal cortex in decision making: a component process account. Ann N Y Acad Sci.

